# A Web-Based Programme for Person-Centred Learning and Support Designed for Preschool Children with Long-Term Illness: A Pilot Study of a New Intervention

**DOI:** 10.1155/2012/326506

**Published:** 2012-12-30

**Authors:** Anna-Lena Hellström, Agneta Simeonsdotter Svensson, Ingrid Pramling Samuelsson, Margaretha Jenholt Nolbris

**Affiliations:** ^1^Institute of Health and Care Sciences, The Sahlgrenska Academy, University of Gothenburg, P.O. Box 457, 405 30 Gothenburg, Sweden; ^2^Urotherapy Department, Queen Silvia Children's Hospital, 416 85 Gothenburg, Sweden; ^3^Centre for Person-Centred Care (GPCC), University of Gothenburg, P.O. Box 100, 405 30 Gothenburg, Sweden; ^4^Department of Education Communication and Learning, University of Gothenburg, P.O. Box 300, 405 30 Gothenburg, Sweden

## Abstract

For children living with long-term illness, school age is a risk period with regard to psychosocial ill health and poor compliance with treatment. There is a need for methods to promote health, well-being, and self-esteem. This study describes a new concept for supporting children, person-centred web-based learning and support, which has been tested in 12 preschool children and incorporates learning about feelings, relationships, and the right to integrity. SKYPE was used for conversations between the child and the web teacher. *Methods*. The programme was developed and tested in two steps. The conversations were tape-recorded and analysed using phenomenography. The questions addressed concerned the quality of the intervention process: accessibility of intervention, learning content and support, and identification of measurable items and patterns. *Findings*. The children found it interesting to communicate with their web teacher using SKYPE. The story about Max and Sara served as a good basis for discussion, and development was found in the learning process. The children were able to talk about relations and feelings and developed an understanding for use in new situations in their daily lives. Items and patterns that are useful for research and documentation were identified, for example, well-being, resources, needs, and wishes.

## 1. Introduction 

Children living with a long-term illness are used to facing problems. The child's family contributes to a feeling of safety and comfort for the child [1]. However, when the child starts school, the parents are not there, and friends and peer groups start to become important to the child, and a feeling of not being like others often arises [1–4]. School age is a risk period with regard to psychosocial ill health and poor compliance with treatment [5–7]. Despite efforts to support children when they face these problems, they are not enough and we need to find new methods of prevention and strengthen the children to feel that they are good enough in order to avoid these situations, given the negative consequences for health. The purpose of the project is to strengthen the child's own resources, self-esteem, and process of learning via a web-based programme.

 Young families are occupied with jobs, school, and leisure activities and do not like to spend too much time at hospitals. Receiving information, being together on the Internet and using a computer program, can today be considered a tool to satisfy the needs of patients and their families for support in daily life. Children and adolescents have good skills in Internet-based technology according to a review investigation [8]. In the field of paediatric/adolescent oncology, this has been tried to manage stress and ill health [9]. Eighteen-year-olds with leukaemia took part in a randomized study to evaluate an interactive programme through which symptoms and signs of ill health could be communicated. In one group that had access to the programme many questions and problems could be resolved at an early stage. In another computer-based study, participatory design has been used to support seriously ill children [10]. The children, aged nine to eleven years, participated with useful ideas during the design process. A new challenge is to design a programme that can be used by children who cannot read or write. Web-based learning and support for preschool children is a new field that needs study. 

 An overall aim of the project is to develop a user-friendly, quality-assured web-based person-centred model for learning and support that can be used in different paediatric long-term or chronic illnesses. The aim of this specific paper is to describe the intervention tested in a pilot study. The questions addressed concern the quality of the intervention process in terms of accessibility, relevant learning content and support, and identification of measurable items and patterns useful for person-centred learning and support. 

## 2. Theoretical Background, Statement and Intervention

### 2.1. Theoretical Background, and Statement

To focus on the child and the child's resources and needs, the model of person-centred care (PCC) is used [11–13]. PCC is explained as focusing on the person and not the illness, and on the patient's experience of his or her situation. The purpose is to understand behaviour and symptoms from the perspective of the individual patient and to make caring support and treatment fit the person's need. To manage this, it is necessary to proceed from knowledge of daily life and priorities of the child with a long-term illness. For each child, resources and needs are being identified and focused on [13]. The project sheds light on health, which is here considered to be related to the individual. Antonovsky's salutognetic model [14] postulates factors contributing to the maintenance of health and well-being. Regarding health as a point on a health ease/dis-ease continuum and movement towards the health end are important. The child's capacity to stay well and even improve health in difficult situations could be based on three factors: (1) comprehensibility, a combination of the ability to assess and understand their situation, (2): (a) meaningfulness, finding meaning to ability to assess and understand their situation, (b) meaningfulness, finding meaning to moving in a health-promoting direction, and (3): manageability, the capability to do so. In the present study, health equates to well-being.

 In order to study the process of learning and how it occurs, the variation theory by Marton and pong will be used as a basis [15]. This theory has its origins in phenomenography, which studies relations between individuals and what will be learned. This theory points out a need for variation in perception of different aspects of a phenomenon. By discerning different aspects of the studied phenomenon, it can be experienced and ultimately understood by the child. Samuelsson and Carlsson [16] have focused on preschool and educational development in their research, with the aim of getting the child to think about, reflect on, and communicate his or her thoughts. Three levels are described. Level 1 signifies that the child's perception of the variations in the teaching is identified and reflected. In level 2, general structures become observable, and in level 3 there is a perception of the child's own learning, what, how, and why the child acts in a certain way and how he or she could act differently. 

 Learning is a central aspect of the study, and the starting point is the child's perspective and experience. The focus is on how the child perceives the content and various objects of learning within it, that is, the meaning that the child constructs for it. For example, when a new theme is introduced in the web programme, it has to be raised and highlighted [17]. In the present study, a pedagogic approach is used to support health and well-being, which may be related to the way the child experiences the problem that he or she deals with in life. The way the child communicates his or her perception/experience is important to the process of learning and the evaluation of the study. The child's perception of different parts of health and well-being can be communicated between the child and the web teacher (here the researcher) to illuminate a variation in the way the content is perceived. This approach to learning is called the development pedagogic and is linked to variation theory adjusted to preschool children [16]. In preschool educational research, it is important to relate playing and learning to each other. Furthermore, playing is a tool for children in communication and cooperation with other children and adults. When playing, the child develops social competence, which is important to the ability to compromise and feel sympathy and empathy [17]. In this project, playing is regarded as an instrument to obtain reflective knowledge [16, 17].

### 2.2. Intervention

The intervention is designed like a virtual preschool for children of four to six years of age and is developed for use on a website platform and SKYPE. This service allows users to communicate by web camera over the Internet. The teaching is built on pictures developed for this project from “see, hear, and do” cartoons used for children with cancer [18]. The child receives the necessary equipment to communicate, a tablet computer, and SKYPE, together with printouts of the pictures.

 The programme starts with an introduction and face-to-face supervision between the researcher/web teacher and the child, which also provides an opportunity for the child and the web teacher to get to know each other.  This procedure helps to overcome cognitive, psychosocial, and language barriers. The children are encouraged to use the programme whenever they wish between the predetermined followups each month. Useful information for parents about communication is accessible on the platform.

**Figure 1:**
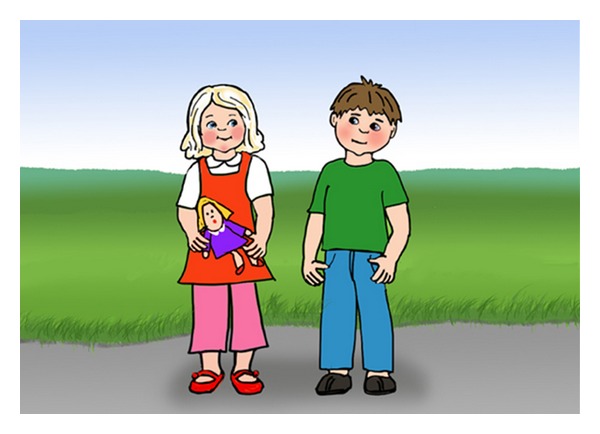
Max and Sara, the children in the intervention. Illustrations by Gunilla Wärnström.

**Figure 2:**
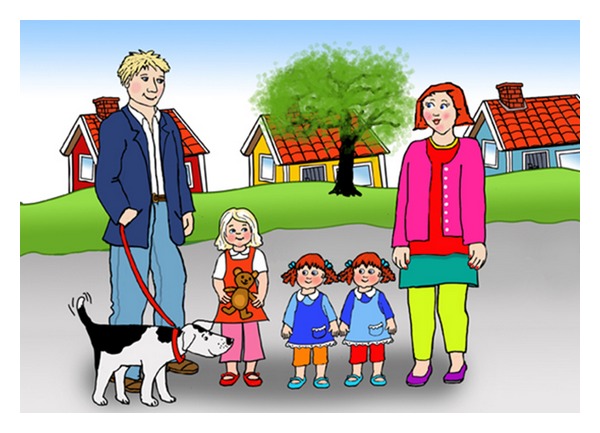
Sara's second family. Illustrations by Gunilla Wärnström.

 On the platform, the preschool web library presents the different sections. There are instructions (a voice talking), sounds, and pictures illustrating different actions. The main topics are relations, feelings, the right of integrity, body function, and basic preschool knowledge. The first section used from the web library is called Max and Sara's families. Max and Sara are the children in the story (Figure 1). In this section, a common range of family constructions is introduced (Figure 2). The purpose of these pictures is to understand the family relation, in general and for the individual, and its meaning/roles. Exercises using numbers, letters, prepositions, and colours are also included. A web teacher instruction is included to guide the teacher to achieve the aim of particular pictures. After the presentation, the first question to discuss could be “how about your family? How many people are in your family?” Depending on the answer, the conversation should develop to achieve the above aim. Resources and needs are identified, and the teacher concentrates on the needs and strengthens the resources. 

**Figure 3:**
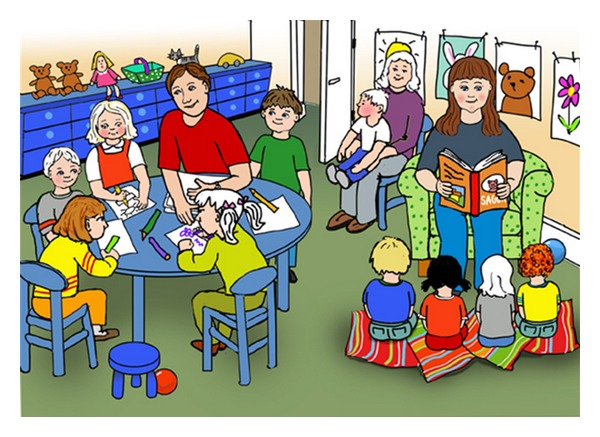
At preschool. Illustrations by Gunilla Wärnström.

**Figure 4:**
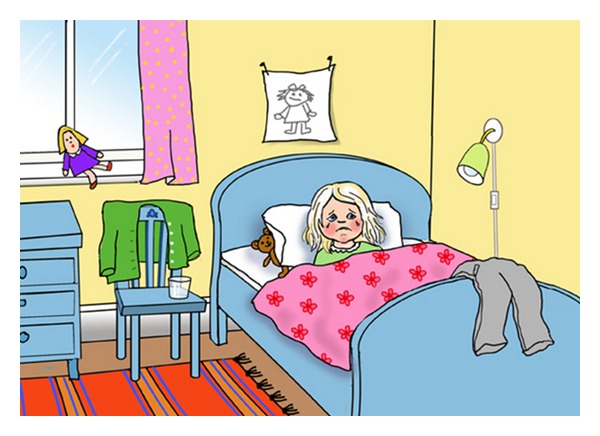
Sara feels ill and has to stay in bed. Illustrations by Gunilla Wärnström.

 The second section, called Max and Sara's preschool, is about the preschool setting and relations outside the family (Figure 3), but content from the first section is still involved and practised if necessary. The purpose of the second section is to understand relations outside the family and their meaning/roles, friendship/friends, and learning/teacher. Another purpose is to get meanings and words for emotions and learn to understand feelings like happiness, fear, and anger. What they feel like and how to recognize different feelings in other people are discussed as are different reasons for feeling like that (Figure 4).

a/How to make friends and social rules are discussed: b/fun things I do/I want to do; c/tedious things; d/paint how it feels when you are angry, sad, happy; g/how does it feel; h/what do you think you can do; i/is it good to show what you feel; j/stories that describe feelings are discussed; k/integrity and the right to say stop are discussed.

 The third section is about the human body. The purpose of this section is to obtain knowledge about the function of the body and reinforce what is healthy. We also discuss what can go wrong, illness and how to treat or compensate for it. This section was not included in the present pilot study.

 The teaching method at the web preschool may vary and, in addition to pictures, there can be quizzes, film sequences, songs, and fairy tales. The design of the platform aims to be attractive and to strengthen health and the child's own resources and to cater to the child's joy of playing. In the pilot study, we mainly used the pictures.

## 3. Methods

### 3.1. Participants and Process

The intervention was pilot tested in two parts in order to ensure the quality of the web preschool before a larger study could be conducted. The first test involved healthy children, three in home settings, and four in a preschool setting. Altogether, seven children, aged three to six years, participated: six boys and one girl. After an introduction, the children took part in the intervention, and the conversation between the web teacher and the child was observed and notes were taken. Between each intervention, the programme was improved and updated according to the results. 

 The second test included five children with recurrent urinary tract problems. All of them were girls aged between four and six years. This time the intervention was tested on the web using SKYPE. This means that the conversation between the child and the web teacher was conducted using a web camera and the Internet.

The introduction was conducted and followed the described intervention. Follow-up conversations on the web platform according to the programme were performed after one and two months. The conversations were tape-recorded and transcribed verbatim.

 The child was encouraged to consider different phenomena in each theme and picture concerning his or her situation and experiences in daily life [16]. The web teacher offered enough time for the child to think about and reflect what happened, what they did, how and why they did so, and what they could do, and furthermore what they wished could happen. The conversation between the child and the web teacher included open questions related to the aim of each part of the intervention. After the introduction, questions could be raised such as: What do you think? Do you recognize this? what is it like in your family (or at preschool)? Tell me! How would you like it to be? Why do you think it is like that? What could you (or Sara, Max) do to feel better? In this second test, the conversation continued and developed following the themes and what was discussed before.

 After the last intervention, at two months, interviews with the parents were also conducted using short open questions on what they thought about the intervention, such as accessibility, content, and their child's interest. These answers were used to support the findings from the child-web teacher conversation.

### 3.2. Analysis and Method

The quality of the intervention process was investigated by analysing the conversation between the child and web teacher in addition to the interviews with the parents. Relevant content in learning and support was investigated by analysing the text. Measurable items and patterns were identified, considering accessibility, person-centring, health, and learning. 

 The method chosen for the analysis was phenomenography [15]. The transcribed text from conversations and interviews was read carefully by the authors. The interpretation from the entirety to the parts was conducted in order to increase understanding of the text. Throughout the process, the parts of the text were first analysed independently by the authors for agreement and then the categories were checked for content to increase the trustworthiness of the analysis. The children's and parents' statements were identified and discriminated. The entirety and the parts were meet and the dependency between them was seen to answer the aim of the study. Room for the outcomes was created for categories describing the responder's different ways of apprehending/experiencing the phenomenon. Similarities in understanding constituted one category. In the present study, two main categories were generated: consequences of playing and consequences of experiences. Each main category consisted of two subcategories: “the child's participation in playing at preschool” and “the child's participation in playing in the family and among friends,” and “feelings related to preschool” and “feelings related to the family.” The criteria for a category were fulfilled according to the method: to have a relation to the phenomenon, a logical relation between categories and as few categories as possible to find the critical variation in data [15]. Representative quotes of statements by the children supported the meaning of the categories and increased trustworthiness. The interviews with the parents, with a few short open questions, were analysed and sorted according to their experience of the intervention, in terms of accessibility, relevant content, and their child's interest.

### 3.3. Ethical Consideration

Informed consent was obtained from the children and the parents. The children and their families were informed that they could interrupt their participation at any time. Confidentiality was ensured. The Regional Ethical Committee for research has approved the project. 

## 4. Results and Discussion

This section describes findings from the web intervention as a method, results from the conversation between the child and web teacher regarding person-centred learning and support, accessibility from the parents' point of view, and items and patterns measurable for health and PCC.

### 4.1. Feasibility and Satisfaction with the Intervention

A web preschool is a new intervention to support children with long-term illness. This study has shown that web-based support in younger children who are not able to read and write is possible. The children found it interesting to communicate with their web teacher on SKYPE, and the story about Sara and Max constituted a good basis for the conversation. The children in the study could find the intervention on the platform and they also received paper copies of the pictures. By using SKYPE, an on-going see-hear conversation was facilitated and the children were able to show the teacher what they liked to illustrate. The teacher for her part was able to watch the child's face and body expressions and respond to them. She could also observe when the child was not focused or too tired to continue. The see-hear pictures were suitable for this type of intervention, giving the child the chance to understand and reflect on the situation. The web teacher was able to vary the theme of the learning process. Similar tools of pictures and photo voice have been used before in information and in support of children face to face [19–22]. The follow-up design allowed the child and teacher to get to know each other, which might have enabled deeper conversation. The phenomenographic method with respect to the development pedagogic method described by Samuelsson and Carlsson was useful for analysis according to the aim [15, 16].

 Altogether, 12 children participated in the pilot study, and five of them took part in the follow-up conversation according to the intervention. The number of participants included was not decided beforehand but was dependent on developing suitable intervention. Between 13 and 60 minutes were spent on each conversation on 22 occasions. The web teachers were two of the authors and had special knowledge of preschool children and the method (M. Jenholt Nolbris, PhD, paediatric nurse and A. Simeonsdotter Svensson, PhD, pedagogue). 

### 4.2. Results Person-Centred Learning and Support

#### 4.2.1. Consequences of Playing

This category describes the child's participation in playing in the family and at a preschool setting. Most of the children's narratives were related to playing, and it was important to participate in the playing. The importance of playing in childhood has been stressed many times before [16, 17, 23, 24] and it was therefore not a surprise that the experiences were expressed through playing. Already in 1963, Erikson [25] described from a psychoanalytic perspective that playing can be used as a therapeutic tool for selfcare in children, which the present intervention will also support in daily life. Children's hospitals today often use play therapy for learning about a procedure. To follow up the given information and solve misunderstandings, procedural play has been used with good results [26].

 In the conversation, the children reflected on situations of which they had experience, and how they thought about these. The child described the pictures he or she had discussed earlier with the web teacher. The teacher affirmed the child by listening to him or her and telling the child that he or she was good at describing. The children did very well with the narration. One reason could be the expectations of the children to respond to the web teacher. Participation by children is stressed as important in the Convention of Children's Right [27]. Children who participated, in planning and decisions at an early age develop an ability to reflect and argue, and this has been shown to facilitate their self-esteem [28]. The latter study has shown that already from the age of two years, children are able to take part in decision-making concerning their health. In the present study, children expressed a need to talk about their experiences; they liked to be addressed and the intervention could contribute to their self-esteem.

**(i) Participating in Playing with Family and Friends at Home**

In this subcategory, playing in the family and together with friends is described. The child related his or her experience at home by reflecting on what he or she had heard about Max and Sara. The follow-up conversation showed a process of developed understanding, and the child was able to draw conclusions from a certain event to a consequence, a next step in the process, “*The best thing is when there is snow and we can throw snowballs at Daddy…Max and Sara go skiing… When there's snow outside, you can go on a small sledge, snow racer or throw snowballs.*”

The children showed that they participated in the situations that arose and played in the family and together with friends. The children could draw logical conclusions from what was possible in different situations, “*I jumped on the trampoline today; it was very cold; you should have shoes on. My feet were cold.*” This quotation has the three steps described by Pramling Samuelsson and Asplund Carlsson: first, the learning objective, second, the general structures became visible to the child, and third, being able to think about one's own learning [16].

Games and playing were regarded as the same thing, but the children knew they could win when playing games. Playing was a common tool to communicate with other children, “*I have been at my friend Johan's house. We usually play games, and the one that is most fun is Mario Kart, a game with two men, one is Luigi and the other one is Mario. You play it on Nintendo. I am very good at it. You can win a cup. It is made of gold and very big.*” Playing as a means of communication has been described earlier in a study of preschool children [29].

**(ii) Participating in Playing at Preschool**

Playing at preschool was something children often talked about. They played together with the other children. This was regarded as the most important thing and proof of participation in the community. The social rules of playing were important; the rules were often set up by the teachers. To be able to participate fully, social and emotional competence is needed, but none of the children who participated in the pilot study had any problem with alienation, “*I was at preschool yesterday, we played outdoors and went down the hill on a bum slider. It did't go that fast; we had to sit and hold on tight.*” This subcategory also had three steps: learning object, general structures, and thoughts about own learning [16]. The following is a quotation by a child describing a game: “…*when you get to 100 you win. I can count to 100. When Max and Sara are at their preschool, they sing the name song. At my preschool we can't sing the name song because there aren't enough children.*” The children talked differently about participating in playing at preschool, and the play could be viewed in different ways. They had in common that they described the playing and its consequences in daily life indoors and out. The children also related to Max and Sara and what they did. These findings pointed out the importance of having a common basis for conversation. The story about Max and Sara involved a theme to talk about that did not necessarily need to be too private.

#### 4.2.2. Consequences of Experiences

This category describes the children's experiences and certain emotions concerning them. The pictures in the story about Max and Sara opened up the conversation about feelings. Feelings in general, fear, death, and sorrow, were mentioned by the children but also situations of joy. Their own and others' experiences are central to children in this category. The children described different feelings related to what they had experienced at home and at preschool. The narratives arose from situations in daily life. The experiences could be more or less positive. According to the intervention, the children were asked by the teacher, “*How did it feel in your body?*” and “*What to do?*”

**(i) Feelings Related to Family**

This subcategory shows how children communicate feelings related to the family and between friends. Different feelings and emotions in relation to family and friends can be experienced as caused by someone. Other people and animals are often mentioned by the child. The children in the study were often able to describe experiences in daily life and put words to their feelings. Their feelings were also mostly reflected followed by a solution of what to do when it happened, “*I became frightened when Daddy scared me. Daddy and Anton scare me and then you run away.*”

Some children had experiences of a pet that had died, and they were mostly related to a feeling of grief. But the children did not necessarily relate death to grief, they could just talk about their experiences of a certain animal, “*I had a guinea pig and his name was Jeppe. He is dead now. He was grey and white. He was naughty and once he bit my finger.*” When the web teacher asked how the child had felt when Jeppe died, the child had answered that she felt sorrow. Feelings could be experienced as just emotions raised and not related to any specific event, “*I feel like it is jittery and it feels like you are a bit shy.*”

Other feelings were related to a well-known event and already reflected, such as when the child got hurt accidentally. Then it is regarded as natural to be upset and sad, “*I feel sad when I get hurt. Yesterday, I fell with my bike. I got a very big hole in my trousers. It did't hurt; I wasn't injured.*” Reflected feelings built on the child's own experience could be put in a new concept that the child could recognize, “*Sara is happy, maybe something fun has happened; a friend had been there.*”

This pilot study indicates that feelings expressed by children may be feelings they know they are allowed to have or can be expected to react to. It means that when a new situation arises when a child is violated/insulted, he or she may not have any words or not even rules to refer to and consequently have a problem telling anyone in a comprehensible way. These feelings could not be reflected in a good way. This stresses the importance of the child's right to integrity and informing and discussing it with the child. Through such discussions in preschool groups, health care professionals and preschool teachers can offer tools to prevent bullying, insults, and sexual abuse.

**(ii) Feelings Related to Preschool**

This subcategory is about feelings related to experiences of the preschool and in the preschool context. Max and Sara's story and the pictures were used as a foundation for the children's own experience to express feelings and reflect on them and also put them into new contexts. They described their reflections as consequences of their experiences, that is, being sad when sad things happened or feeling happy when fun things happened. They showed that they remembered the conversation before and had learned to use words to communicate their feelings. They liked to talk about their experiences of feelings and events when they happened and also to be validated, “*One day Sara didn't want to go to preschool. They had planned to go on an excursion and she was ill. I think she felt sad. I have felt like Sara about not going to preschool; it was boring. It's not fun to go round and round in the forest.*”

It is not always easy to be a child in a big preschool class. It is different from at home and you have to wait for help. Sometimes, the teachers give attention to other children and a particular child may feel lonely, “*Sometimes, I feel lonely.*” In the preschool setting, the child also tried to relate to his or her experiences of situations that allowed them to feel sad, “*Sara is sad, maybe she has got hurt. Once I scraped my knee, then I felt sad*.” Different rules and acceptance or lack thereof have to be discussed at preschool age by professionals to let the child know what is and is not acceptable and further prevent unnecessary suffering. Sometimes, the children experienced that social rules given by the teachers were not being followed. They expressed feelings of worries and sadness, but because they were rules that everyone knew about, they could complain to the teacher, “*You feel sad when someone tells you something not nice/silly. They say…when you are playing and one of them tells you that you are not allowed to join. You feel sad. Then you tell the teacher and the teacher talks to the children who had done the wrong things. Then it starts to be better.*” In our model of web-based learning and support, we have to be aware of the unspoken information of needs and wishes and ask why children act in different ways. Most children like to be liked other children, but they do not know how to express their needs. This was clearly expressed in an earlier published study of boys on treatment with clean intermittent catheterization. They did not tell anyone that they could not cope with the treatment because they were afraid of being teased. They just stopped the treatment and nobody asked why no catheters were used. [2].

The preschool children also liked to feel pride, and they often talked about their skills being validated, “*I can show you a drawing. I am good at drawing.*” This is a good spur in learning and can be used to overcome difficulties in compensating for important needs in health and when expressing wishes [13, 14, 16].

### 4.3. Accessibility of Intervention from the Parents' Point of View

The parents found that the technical communication, including the children's skills in managing the different buttons, was satisfactory, except in one family that occasionally had a slow connection due to weak broadband. The interviews via SKYPE were experienced as exciting. The parents' participation consisted of switching on the computer and SKYPE and being present in the same room when the child communicated with the web teacher. Parents today are web-based communication and they were able to guide their children if necessary. The parents expressed their appreciation of the aim of the intervention to strengthen what was good and not, as is usually done when visiting hospital, what needed to be better.

### 4.4. Items and Patterns Concerning Person-Centring and Health

The interest in learning and the ability of reflected communication were identified as resources of the preschool children who participated in the present study. When the child reflects on the story about Max and Sara by talking about their experience, needs can be identified. The children also practise communicating their feelings and wishes. The web teacher has the opportunity to choose certain themes concerning identified needs and wishes and to concentrate on these in the conversation. The intervention design with follow-up conversation facilitates and ensures a person-centred manner according to the method described by Ekman et al. [13].

Signs of well-being, or a lack thereof, are identified in the conversation, as is support from teachers. Feelings of happiness and pride, finding solutions to problems that arise, and further understanding that actions and reactions from other people are not necessarily due to the child are regarded as signs of well-being. This is in harmony with the definition of health described by Antonovsky [14]. However, this study is too small to confirm all the factors (comprehensibility, meaningfulness, and manageability).

## 5. Conclusions

In this pilot study, we have shown that web-based support is possible for preschool children. Children found it interesting to communicate using SKYPE. The stories about Max and Sara constituted a useful basis for conversations about feelings and participation in playing as an important part of the children's society. The process of learning was supported and shown to be useful: learning something new, reflecting on it, and then using the knowledge in a new context. Items and patterns were identified that were useful for research, considering person-centring, health, and learning. The follow-up interventions after one and two months indicated a development of person-centred learning and support. The individual's resources, needs and intentions were expressed in the present study and communicated in the interaction between the child and the teacher. Support was given as confirmation of the children's narratives and expression of their feelings. Well-being was identified as feelings of happiness, pride, the ability to reach solutions, and understand feelings. Learning about feelings, relationships, and the right to integrity may strengthen self-esteem and well-being and contribute to preventing ill health and isolation at school age. Our findings support the importance of further studying the intervention described in children living with a long-term illness.

## References

[1] Berntsson L, Brydolf M, Berg M, Hellström A-L (2007). School children and adolescents’ perception of health, well-being and participation. *Scandinavian Journal of Caring Sciences*.

[2] Hellstrom A-L, Berg M, Sölsnes E, Holmdahl G, Sillén U (2006). Feeling good in daily life—from the point of view of boys with urethral valves. *Journal of Urology*.

[3] Holmdahl G, Sillén U, Abrahamsson K, Hellström A, Kruse S, Sölsnes E (2007). Self-catheterization during adolescence. *Scandinavian Journal of Urology and Nephrology*.

[4] Taylor RM, Gibson F, Franck LS (2008). A concept analysis of health-related quality of life in young people with chronic illness. *Journal of Clinical Nursing*.

[5] Wilson CJ, Pistrang N, Woodhouse CRJ, Christie D (2007). The psychosocial impact of bladder exstrophy in adolescence. *Journal of Adolescent Health*.

[6] Ebert A, Scheuering S, Schott G, Roesch WH (2005). Psychosocial and psychosexual development in childhood and adolescence within the exstrophy-epispadias complex. *Journal of Urology*.

[7] Af Sandeberg M, Johansson E, Björk O, Wettergren L (2008). Health-related quality of life relates to school attendance in children on treatment for cancer. *Journal of Pediatric Oncology Nursing*.

[8] Lau PW, Lau EY, Wongdel P, Ransdell L (2011). A systematic review of information and communication technology-based interventions for promoting physical activity behavior change in children and adolescents. *Journal of Medical Internet Research*.

[9] Ruland CM, Holte HH, Røislien J (2010). Effects of a computer-supported interactive tailored patient assessment tool on patient care, symptom distress, and patients’ need for symptom management support: a randomized clinical trial. *Journal of American Medical Informatics Association*.

[10] Ruland CM, Starren J, Vatne TM (2008). Participatory design with children in the development of a support system for patient-centered care in pediatric oncology. *Journal of Biomedical Informatics*.

[11] Mead N, Bower P (2000). Patient-centredness: a conceptual framework and review of the empirical literature. *Social Science and Medicine*.

[12] Gambling T, Long AF (2010). The realisation of patient-centred care during a 3-year proactive telephone counselling self-care intervention for diabetes. *Patient Education and Counseling*.

[13] Ekman I, Swedberg K, Taft C (2011). Person-centered care—ready for prime time. *European Journal of Cardiovascular Nursing*.

[14] Antonovsky A (1987). *Unraveling the Mystery of Health How People Manage Stress and Stay Well*.

[15] Marton F, Pong WY (2005). On the unit of description in phenomenography. *Higher Education Research & Development*.

[16] Samuelsson IP, Carlsson MA (2008). The playing learning child: towards a pedagogy of early childhood. *Scandinavian Journal of Educational Research*.

[17] Pramling Samuelsson I, Johansson E (2006). Play and learning—inseparable dimensions in preschool practice. *Early Child Development and Care*.

[18] Gustafsson K, Nolbris M (2006). *See-Hear-Do Pictures. Teaching About Children’s Cancer with Cartoon Tools*.

[19] Nolbris M, Abrahamsson J, Hellström A-L, Olofsson L, Enskär K (2010). The experiences of therapeutic support groups by siblings of children with cancer. *Pediatric Nursing*.

[20] Rollins JA (2005). Tell me about it: drawing as a communication tool for children with cancer. *Journal of Pediatric Oncology Nursing*.

[21] Wang CC, Yi WK, Tao ZW, Carovano K (1998). Photovoice as a participatory health promotion strategy. *Health Promotion International*.

[22] Pridmore P, Bendelow G (1995). Images of health: exploring beliefs of children using draw and write’ technique. *Health Education Journal*.

[23] Milteer RM, Ginsburg KR (2012). The importance of play in promoting healthy child development and maintaining strong parent-child bond: focus on children in poverty. *Pediatrics*.

[24] Howard J, McInnes K The impact of children’s perception of an activity as play rather than not play on emotional well-being.

[25] Erikson E (1963). *Childhood and Society*.

[26] Shields L, Kristensson-Hallström I, Kristjánsdóttir G, Hunter J (2003). Who owns the child in hospital? A preliminary discussion. *Journal of Advanced Nursing*.

[27] http://www2.ohchr.org/english/law/crc.htm.

[28] Shier H (2001). Pathways to participation: openings, opportunities and obligations. *Children & Society*.

[29] Pramling Samuelsson I, Sheridan S (2009). Preschool quality and young children’s learning in Sweden. *International Journal of Child Care and Education Policy*.

